# Drug Use and HIV Infection Status of Detainees in Re-Education through Labour Camps in Guangxi Province, China

**DOI:** 10.3390/ijerph120504502

**Published:** 2015-04-23

**Authors:** Lei Zhang, Lorraine Yap, Joanne Reekie, Wei Liu, Yi Chen, Zunyou Wu, Handan Wand, Tony Butler

**Affiliations:** 1 The Kirby Institute, University of New South Wales, Sydney, NSW 2052, Australia; E-Mails: l.yap@unsw.edu.au (L.Y.); jreekie@kirby.unsw.edu.au (J.R.); Hwand@kirby.unsw.edu.au (H.W.); tbutler@kirby.unsw.edu.au (T.B.); 2 Central Clinical School, Faculty of Medicine, Nursing and Health Sciences, Monash University, Melbourne, VIC 3800, Australia; 3 Research Center for Public Health, School of Medicine, Tsinghua University, 100064 Beijing, China; 4 Division of AIDS/STD, Guangxi Center for Disease Control and Prevention, Nanning 530011, Guangxi, China; E-Mails: lw_gx@126.com (W.L.); chenyi1109@gmail.com (Y.C.); 5 National Center for AIDS/STD Control and Prevention, China CDC, Beijing 102206, China; E-Mail: wuzunyou@chinaaids.cn

**Keywords:** drug use behaviour, incarceration, HIV, re-education through labour camps, China

## Abstract

This study describes HIV disease burden and patterns of drug use before and during incarceration among detainees in Re-education-Through-Labour-Camps (RTLCs) in China. A cross-sectional survey of 576 men and 179 women from three RTLCs was conducted in Guangxi Province, China. Over three-quarters of study participants were detained due to drug-related offences. Over half of the women (n = 313, 54.3%) and two-thirds of men (n = 119, 66.5%) had been previously been incarcerated in a compulsory detoxification treatment centre (CDTC), and around one-third (men n = 159, 27.6%; women n = 50, 27.9%) in a RTLC. Of those surveyed, 49 men (8.5%) and one (0.6%) woman reported ever using drugs while in a CDTC and/or RTLC. Previous incarceration in CDTCs and RTLCs were associated with HIV infection among both male (*OR =* 2.15 [1.11–4.15]) and female (*OR =* 3.87 [1.86–9.04]) detainees. Being married/cohabiting with a partner (*OR =* 0.53, [0.30–0.93]) and being employed (*OR* = 0.46, [0.22–0.95]) were associated with a reduced odds of HIV infection among male detainees. A significant proportion of RTLC detainees had a history of drug use and a limited number of inmates had used illegal substances whilst in custody. Repeat incarcerations in CDTCs/RTLCs were associated with higher risks of HIV infection.

## 1. Introduction

Drug abuse has become widespread in China, and incarceration is increasingly common among people who use and inject drugs [1]. By the end of 2011, there were 1.8 million registered drug users in China, about 171,000 drug users were sent by the police to mandatory or compulsory detoxification treatment centres for rehabilitation, and approximately 97,000 drug users were in community-based rehabilitation centres [2]. Worldwide, rates of human immunodeficiency virus (HIV) and hepatitis C virus (HCV) infection in prisoner populations are much higher than those found in the general population, and this has been attributed largely to sharing contaminated injecting equipment [3,4]. Studies also report that some injecting drug users continue to inject whilst in custody [5,6,7,8,9,10,11]. However, few jurisdictions internationally provide clean injecting equipment or methadone maintenance treatment to those in custody [12].

### Detention Facilities in China

Three main types of detention facilities can be distinguished in China: (1) *jiedusuo—*mandatory or compulsory detoxification treatment centres (CDTCs); (2) *laojiaosuo* or re-education through-labour-camps (RTLCs); (3) *laogaisuo* or reform through labour—the general prison system which includes prisons, prison farms and labour camps. CDTCs were established in 1989 after the Chinese State Council issued a Mandatory Drug Treatment Methods administrative decree granting the Ministry of Public Security the right to establish mandatory or compulsory drug detoxification centres for drug users. In parallel, the Ministry of Justice also has the right to establish CDTCs in a similar structure by its own or in cooperation with the Ministry of Public Security. All CDTCs detainees were drug users. They underwent mandatory detoxification with minimal medical interventions, including opioid substitution therapy. Eligible HIV-positive detainees received ART on sites. In 2003, the Chinese State Council issued the Re-education-Through-Labour Drug Detoxification Regulations administrative decree, to develop drug treatment programs in RTLCs and accept transfers of drug users from the public security departments [13,14]. However, since the promulgation of the Drug Prohibition Law of the People’s Republic of China, drug users without major offences were recommended to be sent to CDTCs prior to RTLCs [15]. The legal articles and regulations on compulsory drug rehabilitation are regularly updated and disseminated by the State Council and the Ministry of Public Security of China [16].

RTLCs and the *laogaisuo* are administered by the Ministry of Justice and related provincial and local departments, while the CDTCs are operated by the Ministry of Public Security and related departments. CDTCs detain individuals sentenced for drug use, and drug possession whereas RTLCs contain a mix of both drug users and non-drug users imprisoned for minor crimes such as drug use and possession, petty theft, and sex work activities while those in the *laogaisuo* tend to have committed more serious crimes, such as homicide and political corruption. Most drug users in RLTCs will have already spent time in a CDTC prior to entering the RTLC. Drug treatment in these facilities is minimal [17] with detainees receiving moral, legal, and drug-related health education in CDTCs and RTLCs [14].

In China, there are estimated to be 1.5–3.0 million injection drug users [18]. Data from sentinel surveillance sites indicates that the HIV prevalence rate has fallen among people who inject drugs (PWID) from 9.3% in 2009 to 6.4% in 2011. Around one-third of PWID use contaminated injecting equipment [19] and intravenous drug use is the second most common cause of HIV transmission in China [17]. Of an estimated 780,000 people living with HIV/AIDS (PLWA) in China, 28.4% are thought to have been infected by intravenous drug use [19]. The numbers of people using new types of drugs (amphetamine-type stimulants and ketamine) continues to grow and accounted for 27% of drug users by 2009 [20]. China’s HIV epidemic is rapidly spreading into the general population through heterosexual (46.5%) and homosexual contact (17.4%) [19]. The proportion of new infections from heterosexual contact increased from 33.1% in 2006 to 76.3% by 2011, and homosexual contact from 2.5% in 2006 to 13.7% in 2011 ([19], pp. 27, 38).

While studies have reported on detainees prior to incarceration in mandatory or CDTCs [21,22,23,24] none have reported on the characteristics of drug users in the RTLCs in China. This study presents data on drug users in RTLCs and compares the characteristics of RTLC detainees who have previously been in the CDTCs with those who have not, and compares the characteristics of HIV positive and HIV negative injection drug users in the RTLC.

## 2. Methods

The study was conducted in three RTLCs in Guangxi, a Southwest China province known to be on the drug-trafficking route from bordering Vietnam, and has a high HIV prevalence among its drug users [25]. Interviewer-administered face-to-face questionnaire interviews were conducted with detainees in prison sports grounds, factories, and outside the gates of the labour camps. Drug use is prohibited in RTLCs. Prison officers were not present during the interviews. Men and women, 18 years and over, were required to sign an informed consent form before they could take part in the study.

### 2.1. Selection of Study Sites

Three out of seven RTLCs in the region (two male and the only female RTLC in the province) agreed to participate in the survey. From our preliminary investigations, we did not detect any major differences between the RTLCs in the region in terms of prisoners’ profile and characteristics.

In the three labour camps, large accommodation multi-story building blocks (with shared rooms and dormitories) were randomly selected for the study and every prisoner living in these selected buildings was invited to participate in the survey. We initially proposed a cross-sectional, individually randomised survey design. However, prison authorities were concerned about choosing individuals from a randomly generated list, believing that this could create conflict and suspicion between those selected and not selected within the camp building blocks. Instead, prison authorities suggested that it would be more feasible to randomise by prison block, since they could more easily control the flow of prisoners coming in and out of the buildings for the survey and ensuring that each area remained secure.

In each RTLC, most HIV-infected prisoners were accommodated in a separate block on the RTLC grounds so that HIV antiretroviral therapy could be more efficiently delivered and to protect HIV-negative prisoners from HIV transmission. All prisoners housed in the separate facilities for HIV-positive prisoners were sampled to ensure greater anonymity when reporting the findings. This administrative process was dependent on HIV status for placement of detainees and repeated across the labour camp sites in the study. The building number was part of the survey and not blinded to the interviewers and interviewees.

### 2.2. Training

The interviews were conducted by 19 medical university students in three RTLCs in Guangxi province during March and May 2011, under the supervision of Guangxi Center for Disease Control and Prevention (CDC). Prior to the survey, interviewers were trained to conduct the surveys. They were asked to deliver informed consent prior to the survey. They were directed to exclude participants who were aged below 18 years and those who spoke local dialect only and unable to understand Mandarin. Interviewers were trained to observe participants, particularly repeated responses of refusing to answer questions and participant’s body language. The trainer (LY) provided experience of Australian prisoners and in-depth interviews which were stopped midway whereby participants were asked if they felt the questions too difficult or if they wanted to continue. The importance of collecting good quality data was stressed and highly dependent on the voluntarily participation of respondents in the study was emphasised to interview staff. At the end of the survey, twenty-nine prisoners aged between 14 and 17 years were excluded from participating in the survey as they were under 18 years old. No participants were excluded because of severe mental health issues or were non-Mandarin speakers.

### 2.3. Sampling

In RTLC One, 20% men (252 out of 1250 registered prisoners in the RTLC) from seven buildings and one HIV rehabilitation unit which comprised in total 62 cells were surveyed. In RTLC Two, 22% of men (324 out of 1500 estimated prisoners) from four buildings and one HIV rehabilitation unit which comprised in total 41 rooms were surveyed. In RTLC 3 (the provinces’ only women’s facility), 33% women (179 out of 540 estimated women) from three buildings and one HIV rehabilitation unit, comprising a total of 26 rooms participated in the survey.

The survey response rate was 100%. This was likely due to prisoners not wishing to go against the group dynamics and conforming to building/dormitory leaders’ instructions and their colleagues’ team expectations and living in the same building. The behaviours are also an artefact of how labour camps are organised. Individuals are placed in workgroups of cadres who live, work, eat and sleep together and are managed by a cadre team leader, also a detainee. Labour camp activities are usually completed in a team. A small remuneration was offered to inmates for participating in the survey.

### 2.4. Survey Questions

Prior to the survey, Chinese epidemiologists from the Center for Disease Control and Prevention (Gunagxi) conducted preliminary investigations and met with labour camp administration and health staff to understand how the labour camps operated and to discuss practical issues related to how the survey could be conducted in the labour camps. In addition, they completed qualitative research interviews with former and current labour camp detainees, men and women, to develop the survey instrument. The survey was translated into Mandarin and back translated to English a number of times during its development phase to ensure the meanings were the same in both languages. The translation was done by study investigators who were fluent in both languages.

We collected the following information from participants: socio-demographics; knowledge and attitudes towards tuberculosis, sexually transmissible infections, HIV and HCV; general health status; HIV testing history; methadone maintenance treatment; mental health; sexual health behaviours; drug use and injecting behaviours; body piercing; tattooing; other risk behaviours; quality of health services and health service delivery inside labour camps; and respondents’ evaluation of their responses to the survey.

### 2.5. Validation of Self-Reported HIV Status

Medical records were available for 429 (57%) detainees who participated in the survey. These included the results of mandatory HIV tests for detainees when they registered in the facility. Those with a positive diagnosis receive post-test counselling and accommodated in a separate building. The medical records were used to verify the self-reported HIV status of these detainees. Of the 429 detainees, 60 detainees (14%) were verified as HIV positive from their medical records; and of these, 58 had confirmatory tests at a CDC laboratory and the remaining two were referrals. All of the negative test results had been conducted within the individual RTLCs and were not sent to the CDC for confirmatory testing. We found a high degree of concordance between the available HIV test results and the self-reported HIV status (Kappa = 0.92, *p* < 0.0001). HIV test results were available for the majority of women who completed the survey (174, 97%), all their medical records matched with their self-reported HIV status. Among the 255 (44%) male detainees with HIV test results available, only eight detainees had discordant results whereby their HIV results did not match their self-reported HIV status (89% sensitivity, 98% specificity, positive predictive value 89%, negative predictive value 98%, kappa = 0.87, *p* < 0.0001). The small record discordance implies a high level of consistency in the survey responses. In addition to the medical records validation, detainees self-reported HIV status was also verified by their placement in the HIV units inside the labour camps. Discussions with labour camp and medical staff revealed that every new detainee in the labour camp has to undergo a blood test for HIV on entry (100%). This was validated by our survey which found that 96% of 755 participants who endorsed that they had “ever had a blood test in a labour camp”.

### 2.6. Data Analysis

A unique identification number was given to each participant to ensure that they could not be identified from the survey forms. HIV-infected and non-infected detainees were compared using chi-squared tests by demographic characteristics and offence history, drug use and injecting and needle/syringe sharing behaviours. Where the expected number of events in a category was less than five, Fisher’s exact test was used. All statistical analysis was performed using STATA/SE 12.0.

### 2.7. Ethics

Ethics approval was obtained from the Institutional Review Board at the National Center for AIDS/STD Control and Prevention, China CDC, Ministry of Health, and the Human Research Ethics Committee (HREC) at the University of New South Wales (HREC9125). Permission was also granted to conduct the study from the Bureau of Health and Re-education-Through-Labour Administration in the region. Participation of the study was voluntary and detainees could choose to terminate the interview at any point of the study.

## 3. Results

### 3.1. Demographic Characteristics

We surveyed a total of 755 detainees; 576 men and 179 women in custody in three RTLCs in Guangxi, China. Participants had a median age of 33 years (range: 27–39 years). Male detainees were more likely to be single (49.0% *vs.* 38.0% women), better educated (15.5% *vs.* 7.3% women) and employed prior to prison (51.6% *vs.* 41.3% women).

### 3.2. Drug Use and Sexual Behaviours in Community

Of the total population surveyed, 433 (75.2%) men and 149 (83.2%) women were drug users (*i.e.*, they had used illicit drugs in 12 months prior to incarceration) in the community (Table 1) with heroin the most commonly used drug (97.5% men; 98.7% women, Table 2) followed by ketamine (46.7% men *vs.* 43.0% women), and diazepam (42.0% men *vs.* 34.2% women).

**Table 1:** Sociodemographics characteristics of labour camp detainees, male and female.

Items	Total (n = 755)	Males (n = 576)	Females (n = 179)	Gender Comparison Chi-2 Test, *p*-Value
**Sociodemographic characteristics**				
**Age Group**				
*<25*	103 (13.6%)	88 (15.3%)	15 (8.4%)	χ^2^ = 7.25, *p* = 0.06
*25–34*	328 (43.4%)	246 (42.7%)	82 (45.8%)	
*35–44*	246 (32.6%)	188 (32.6%)	58 (32.4%)	
*45+*	78 (10.3%)	54 (9.4%)	24 (13.4%)	
**Marital Status**				
*Single (never married)*	350 (46.4%)	282 (49.0%)	68 (38.0%)	χ^2^ = 11.45, *p* = 0.01
*Married/Cohabiting*	315 (41.7%)	236 (41%)	79 (44.1%)	
*Divorced/Separated/Widow*	89 (11.8%)	57 (9.9%)	32 (17.9%)	
*Missing/Unknown*	1 (0.1%)	1 (0.2%)	0 (0.0%)	
**Education**				
*Junior high* *school* *and below*	653 (86.5%)	487 (84.5%)	166 (92.7%)	χ^2^ = 8.24, *p* = 0.02
*Senior high* *school* *and above*	102 (13.5%)	89 (15.5%)	13 (7.3%)	
**Employment**				
*No*	384 (50.9%)	279 (48.4%)	105 (58.7%)	χ^2^ = 5.61, *p* = 0.02
*Yes*	371 (49.1%)	297 (51.6%)	74 (41.3%)	
**Sexual behaviours in community**				
**Type of last sexual partner**				
*Regular*	607 (80.4%)	449 (78.0%)	158 (88.3%)	χ^2^ = 14.32, *p* < 0.01
*Casual*	75 (9.9%)	66 (11.5%)	9 (5.0%)	
*Commercial*	50 (6.6%)	38 (6.6%)	12 (6.7%)	
Missing/No response	23 (3.0%)	23 (4.0%)	0 (0%)	
**Condom usage with last sexual partner**				
*No*	571 (78%)	445 (80.5%)	126 (70.4%)	χ^2^ = 8.01, *p* = 0.01
*Yes*	161 (22%)	108 (19.5%)	53 (29.6%)	
**Sell sex in last 12 months**				
*No*	685 (90.7%)	552 (95.8%)	133 (74.3%)	χ^2^ = 137.09, *p* < 0.01
*Yes*	51 (6.8%)	5 (0.9%)	46 (25.7%)	
Missing/No response	19 (2.5%)	19 (3.3%)	0 (0%)	
**Condom usage with client in last sexual act**				
*No*	23 (45.1%)	3 (60.0%)	20 (43.5%)	χ^2^ = 10.61, *p* = 0.01
*Yes*	27 (52.9%)	1 (20.0%)	26 (56.5%)	
Missing/No response	1 (2%)	1 (20.0%)	0 (0%)	
**Buy sex in last 12 months**				
*No*	551 (73.1%)	373 (64.9%)	178 (99.4%)	χ^2^ = 82.93, *p* < 0.01
*Yes*	178 (23.6%)	177 (30.8%)	1 (0.6%)	
Missing/No response	25 (3.3%)	25 (4.3%)	0 (0.0%)	
**Condom usage with sex worker in last sexual act**				
*No*	78 (43.8%)	78 (44.1%)	0 (0.0%)	χ^2^ = 0.78, *p* = 0.38
*Yes*	100 (56.2%)	99 (55.9%)	1 (100.0%)	
**Drug use history in community**				
**Ever used drugs community**				
*No*	173 (22.9%)	143 (24.8%)	30 (16.8%)	χ^2^ = 5.25, *p* = 0.07
*Yes*	582 (77.1%)	433 (75.2%)	149 (83.2%)	
**Ever injected illicit drugs in the community**				
*No*	113 (19.4%)	93 (21.5%)	20 (13.4%)	χ^2^ = 5.81, *p* = 0.06
*Yes*	469 (80.6%)	340 (78.5%)	129 (86.6%)	
**Re-used someone else’s needle and syringes at last injection**				
*No*	407 (86.8%)	293 (86.2%)	114 (88.4%)	χ^2^ = 0.38, *p* = 0.54
*Yes*	62 (13.2%)	47 (13.8%)	15 (11.6%)	
**History of incarceration (CDTC & RTLC) and methadone program**				
**Incarceration in a CDTC**				
*Never*	323 (42.8%)	263 (45.7%)	60 (33.5%)	χ^2^ = 9.06, *p* = 0.01
*Once*	157 (20.8%)	110 (19.1%)	47 (26.3%)	
≥2 times	275 (36.4%)	203 (35.2%)	72 (40.2%)	
**Incarceration in a RTLC**				
*First time*	546 (72.3%)	417 (72.4%)	129 (72.1%)	χ^2^ = 0.01, *p* = 0.91
≥2 times	209 (27.7%)	159 (27.6%)	50 (27.9%)	
**Current offence**				
*Drug related ^1^*	572 (75.8%)	425 (73.8%)	147 (82.1%)	χ^2^ = 5.22, *p* = 0.02
*Non drug related ^2^*	183 (24.2%)	151 (26.2%)	32 (17.9%)	
**Ever been on a methadone program**				
*No*	549 (72.7%)	435 (75.5%)	114 (63.7%)	χ^2^ = 9.87, *p* < 0.01
*Yes*	206 (27.3%)	141 (24.5%)	65 (36.3%)	
**Drug use during imprisonment**				
**Ever used drugs inside a CDTC or RTLC**				
*No*	705 (93.4%)	527 (91.5%)	178 (99.4%)	χ^2^ = 14.01, *p* < 0.01
*Yes*	50 (6.6%)	49 (8.5%)	1 (0.6%)	
**Ever injected illicit drugs inside a CDTC or RTLC**				
*No*	29 (59.2%)	28 (59.6%)	1 (50.0%)	χ^2^ = 0.07, *p* = 0.79
*Yes*	20 (40.8%)	19 (40.4%)	1 (50.0%)	
**Re-used someone else’s needle and syringe at last injection inside a CDTC or RTLC**				
*No*	12 (60%)	11 (57.9%)	1 (100.0%)	χ2 = 0.70, *p* = 0.40
*Yes*	8 (40%)	8 (42.1%)	0 (0.0%)	

**^1^** selling or using drugs; **^2^** political reasons, theft, sex work and other offences.

**Table 2:** The major drug types among those who had ever used drugs in community.

Drug Type	Total (n = 582)	Males (n = 433)	Females (n = 149)
*Heroin*	569 (97.8%)	422 (97.5%)	147 (98.7%)
*Ketamine*	266 (45.7%)	202 (46.7%)	64 (43.0%)
*Diazepam*	233 (40.0%)	182 (42.0%)	51 (34.2%)
*Ecstasy*	176 (30.2%)	128 (29.6%)	48 (32.2%)
*Cannabis*	107 (18.4%)	87 (20.1%)	20 (13.4%)
*Methamphetamine*	98 (16.8%)	79 (18.2%)	19 (12.8%)
*Opium*	56 (9.6%)	50 (11.5%)	6 (4.0%)
*Other*	101 (17.4%)	82 (18.9%)	19 (12.8%)

Approximately two-thirds (63.2%) of those who reported drug use in the community had used two or more different types of drugs in their lifetime. Among drug users, 340 (78.5%) men and 129 (86.6%) women had injected drugs in the community with 47 (13.8%) men and 15 (11.6%) women reported they had re-used someone else’s needle and syringe at the last injection.

Regular partners accounted for 78.0% (449) and 88.3% (158) of sex partners among male and female detainees at their last sex in community, whereas 6.6% (38) men and 6.7% (12) women reported having sex with a commercial sex partner. Notably, 30.8% (177) men and 25.7% (46) of women detainees reported soliciting and selling sex in the last 12 months before incarceration. Among these, only 55.9% (99) men and 56.5% (26) women reported using a condom at their last sexual contact.

### 3.3. Incarceration and Methadone Maintenance Treatment History

Of the 755 labour camp detainees, 313 (54.3%) men and 119 (66.5%) women had ever been sent to a CDTC (Table 1). Of these, 203 (64.8%) men and 72 (60.5%) women had been at least two or more times. In contrast, 417 (72%) men and 129 (72%) women surveyed said that it was their first time inside a RTLC. The most common offences which led to their imprisonment in the RTLC were drug-related (73.8% men and 82.1% women). One quarter of men (24.5%, 141) and 36.3% (65) women reported they had been on methadone maintenance treatment (MMT) before they entered the labour camps. However, no detainees were currently on a MMT program as this treatment was not available at the RTLCs.

### 3.4. Drug Use during Imprisonment

Of those surveyed, 49 men (8.5%) and 1 (0.6%) woman reported ever using drugs while in a CDTC and/or RTLC. Of these, 19 (36%) men and one woman had injected drugs whilst incarcerated in a CDTC and/or RTLC, four of whom were HIV infected. Among those who reported having injected while incarcerated, eight reported re-using someone else’s needle and syringe the last time they injected with three detainees reporting they had re-used someone’s needles and syringes (Table 1).

### 3.5. HIV Prevalence and Associated Factors

A total of 47 (8.2%) men and 25 (14.0%) women reported that they had been tested and informed by a physician about their positive HIV infection status. Among male detainees, being married/cohabiting with a partner (multivariate regression *OR =* 0.53, [0.30–0.93]), being employed (*OR* = 0.46, [0.22–0.95]) were associated with a reduced odds of HIV infection, whereas being incarcerated in a RTLC more than once was associated with an increased odds of being HIV positive (*OR =* 2.15 [1.11–4.15]) (Table 3). Among women, the odds of HIV infection among those incarcerated in a CDTC multiple times was 3.87 (1.86–9.04) times higher than those who had never been incarcerated (Table 4).

**Table 3:** Self-reported HIV prevalence and associated factors among male detainees in RTLC.

Items	Total Counts (n)	HIV Prevalence (%)	Bivariate Reg. OR (95% CI)	Multivariate Reg. OR (95% CI)
**Sociodemographic characteristics**				
**Age Group**				
*<25*	88	2.3%	ref	
*25–34*	245	7.3%	3.41 (0.77–15.01)	
*35–44*	188	9.0%	4.27 (0.97–18.93)	
*45+*	54	7.4%	3.44 (0.61–19.46)	
**Marital Status**				
*Single (never married)*	281	10.0%	ref	
*Married/Cohabiting*	236	4.7%	0.44 (0.21–0.91) *	0.53 (0.3–0.93) *
*Divorced/Separated/Widow*	57	3.5%	0.33 (0.08–1.42)	
*Missing/Unknown*	1	0.0%	--	
**Education**				
*Junior high and below*	485	8.2%	ref	
*Senior high and above*	89	1.1%	0.13 (0.02–0.93) *	
**Employment**				
*No*	279	10.8%	ref	
*Yes*	296	3.7%	0.32 (0.16–0.65) **	0.46 (0.22–0.95) *
**Sexual behaviours in community**				
**Type of last sexual partner**				
*Regular*	449	5.3%	ref	
*Casual*	66	9.1%	1.77 (0.7–4.51)	
*Commercial*	38	26.3%	6.32 (2.76–14.52) **	
Missing/No response	22	4.5%	0.84 (0.11–6.54)	
**Condom usage with last sexual partner**				
*No*	445	7.0%	ref	
*Yes*	108	8.3%	1.21 (0.56–2.63)	
**Sold sex in last 12 months**				
*No*	552	7.2%	ref	
*Yes*	5	0.0%	1.15 (0.06–21.18)	
Missing/No response	18	5.6%	0.75 (0.1–5.8)	
**Condom usage with client in last sexual act**				
*No*	3	0.0%	ref	
*Yes*	1	0.0%	*a*	
Missing/No response	1	0.0%	*a*	
**Bought sex in last 12 months**			**	
*No*	373	5.1%	ref	
*Yes*	177	10.2%	2.11 (1.08–4.13) *	
Missing/No response	25	16.0%	3.55 (1.11–11.37) *	
**Condom usage with sex worker in last sexual act**				
*No*	78	15.4%	ref	
*Yes*	99	6.1%	0.35 (0.13–0.99) *	
**Drug use history in community**				
**Ever used illicit drugs in the community**				
*No*	142	0.0%	ref	
*Yes*	432	9.5%	40.57 (2.48–664.37) **	
**Ever injected illicit drugs in the community**				
*No*	90	3.3%	ref	
*Yes*	339	11.2%	3.66 (1.1–12.15) *	
**Re-used someone else’s needle and syringes at last injection**				
*No*	291	10.0%	ref	
*Yes*	47	19.1%	2.14 (0.94–4.87)	
**History of incarceration (CDTC & RTLC) and methadone program**				
**Incarceration in a CDTC**				
*Never*	263	4.2%	ref	
*Once*	110	5.5%	1.32 (0.48–3.67)	
*≥2 times*	202	11.9%	3.09 (1.48–6.47) **	
**Incarceration in a RTLC**				
*First time*	417	4.8%	ref	
*≥2 times*	158	13.3%	3.04 (1.6–5.78) **	2.15 (1.11–4.15) *
**Current offence**				
*Non drug related*	151	0.7%	ref	
*Drug related*	424	9.4%	15.63 (2.13–114.68) **	
**Ever been on a methadone maintenance program**				
*No*	435	5.5%	ref	
*Yes*	140	12.1%	2.37 (1.23–4.55) **	
**Drug use during imprisonment**				
**Ever used illicit drugs inside a CDTC or RTLC**				
*No*	525	6.9%	ref	
*Yes*	49	10.2%	1.54 (0.58–4.13)	
**Ever injected illict drugs inside a CDTC or RTLC**				
*No*	28	0.0%	ref	
*Yes*	19	21.1%	16.55 (0.84–327.95)	
**Re-used someone else’s needle and syringe at last injection**				
*No*	11	9.1%	ref	
*Yes*	8	37.5%	7.67 (0.49–73.45)	

**^a^** insufficent statistical power due small sampling size; * *p* < 0.05; ** *p* < 0.01.

**Table 4:** Self-reported HIV prevalence and associated factors among female detainees in RTLC.

Items	Total Counts (n)	HIV Prevalence (%)	Bivariate Reg. OR (95% CI)	Multivariate Reg. OR (95% CI)
**Sociodemographic characteristics**				
**Age Group**				
*<25*	15	13.3%	ref	
*25–34*	82	18.3%	1.46 (0.3–7.14)	
*35–44*	58	13.8%	1.04 (0.2–5.5)	
*45+*	24	0.0%	0.13 (0.01–2.82)	
**Marital Status**				
*Single (never married)*	68	13.2%	ref	
*Married/Cohabiting*	79	15.2%	1.17 (0.46–2.98)	
*Divorced/Separated/Widow*	21	19.0%	0.94 (0.27–3.3)	
*Missing/Unknown*	0	0.0%	--	
**Education**				
*Junior high and below*	166	13.9%	ref	
*Senior high and above*	13	15.4%	1.13 (0.24–5.43)	
**Employment**				
*No*	105	19.0%	ref	
*Yes*	74	6.8%	0.31 (0.11–0.86)	
**Sexual behaviours in community**				
**Type of last sexual partner**				
*Regular*	158	15.2%	ref	
*Casual*	9	0.0%	0.34 (0.02–6.04)	
*Commercial*	12	8.3%	0.51 (0.06–4.11)	
**Condom usage with last sexual partner**				
*No*	126	13.5%	ref	
*Yes*	53	15.1%	1.14 (0.46–2.83)	
**Sold sex in last 12 months**				
*No*	133	12.8%	ref	
*Yes*	46	17.4%	1.44 (0.57–3.59)	
**Condom usage with client in last sexual act**				
*No*	20	15.0%	ref	
*Yes*	26	19.2%	1.35 (0.28–6.47)	
**Bought sex in last 12 months**				
*No*	180	13.9%	ref	
*Yes*	1	0.0%	2.03 (0.08–51.28)	
**Condom usage with sex worker in last sexual act**				
*No*	0	--	ref	
*Yes*	1	0.0%	*a*	
**Drug use history in community**				
**Ever used illicit drugs in the community**				
*No*	30	0.0%	ref	
*Yes*	149	16.8%	10.4 (0.62–175.58)	
**Ever injected illicit drugs in the community**				
*No*	19	5.3%	ref	
*Yes*	129	18.6%	4.11 (0.52–32.34)	
**Re-used someone else’s needle and syringes at last injection**				
*No*	113	12.4%	ref	
*Yes*	15	66.7%	14.14 (4.21–47.46) **	
**History of incarceration (CDTC & RTLC) and methadone program**				
**Incarceration in a CDTC**				
*Never*	60	3.3%	ref	
*Once*	47	6.4%	1.98 (0.32–12.35)	
*≥2 times*	72	27.8%	11.15 (2.49–50.04) **	3.87 (1.86–8.04) **
**Incarceration in a RTLC**				
*First time*	129	12.4%	ref	
*≥2 times*	50	18.0%	1.55 (0.64–3.78)	
**Current offence**				
*Non drug related*	32	0.0%	ref	
*Drug related*	147	17.0%	11.24 (0.67–189.39)	
**Ever been on a methadone maintenance program**				
*No*	114	12.3%	ref	
*Yes*	65	16.9%	1.46 (0.62–3.43)	
**Drug use during imprisonment**				
**Ever used illicit drugs inside a CDTC or RTLC**				
*No*	178	14.0%	ref	
*Yes*	1	0.0%	2.01 (0.08–50.62)	
**Ever injected illicit drugs inside a CDTC or RTLC**				
*No*	1	--	ref	
*Yes*	0	--	^a^	
**Re-used someone else’s needle and syringes at last injection**				
*No*	--	--	ref	
*Yes*	--	--	*a*	

**^a^** insufficent statistical power due small sampling size; ** *p* < 0.01.

## 4. Discussion

This study represents one of the few health surveys to have been conducted in labour camps in China and is important in describing the level of need of those in these settings with problem drug use. Drug use in RTLCs and CDTCs is uncommon. We found that only a small proportion of RTLC detainees (6.6%) (50/755) had ever used drugs in custody, and 2.5% (19/755) had ever injected illicit drugs whilst incarcerated in a CDTC and/or RTLC. In China, it appears that rates of injecting drug use in custody are lower than have been previously reported by other international studies of prisons. In Australia, 50% had ever injected inside prison [26]. In Iran, 79% of prisoners reported using drugs, and 6% reported injecting drugs during incarceration [8]. In Canada, 11% of prisoners had injected drugs in the last 12 months whilst in custody [5].

During the survey period, detainees reported that they had been sentenced in the RTLCs mainly for drug related offences (73.8% men, 82.1% women). Many were repeat drug offenders, and most had previously been incarcerated in the CDTC (54.2% men, 67.3% women) prior to entering the RTLC. Being repeatedly incarcerated in confined settings, such as CDTC and RTLC was associated with an increased risk of HIV infection for both male and female detainees. We speculate that this may be due to the inability to access harm-reduction interventions in punitive confinements which leads to subsequent cycles of repeat relapse after release and police arrest of drug users. The lack of coordination between the ministries of justice and health is a major barrier for implementing harm-reduction programs for drug using detainees. The continuous use of or relapse to use drugs in turn substantially increase the risk of HIV infection. In contrast, male detainees who were married/cohabiting with a regular partner and employed in the community had a lower risk of HIV infection. This indicates that a stable family and social network may reduce addictive drug use and hence the risk of acquiring HIV [27]. These findings suggest that it is important to provide appropriate treatment whilst in prison and also to have effective post-release support to prevent relapse to drug use.

RTLCs provide education for inmates on preventing HIV and HCV. In addition to basic primary health care, antiretroviral therapy is also available to eligible (CD4 T cell counts < 350 cells/mm^3^) HIV patients according to the national ‘Four Free One Care’ policy. Most RTLCs, however, do not routinely offer opioid substitution therapy such as MMT to reduce injecting and sharing behaviours between inmates. These data suggest that around two-thirds of heroin users have never accessed MMT programs in the community prior to incarceration. Further, the one-quarter of the detainees who had access to MMT prior to entering labour camps were unable to continue their treatment inside the labour camps. Evidence on opioid substitution therapy in prison suggests that it reduces injecting drug use and needle and syringe sharing in prison, recidivism, and post-release heroin use [22,23,24,25,26]. Currently, the national MMT program in China treats over 340,000 drug users across the country, corresponding to a 30% program coverage among the drug users [28]. RTLCs could potentially be a setting to offer MMT as a means to prevent repeat offending for drug use charges. Most prisoners in RTLCs have been detained on drugs-related charges. Given the fact that four of the nineteen PWID detainees were HIV-infected, providing the harm-reduction services, in addition to the existing antiretroviral therapy, to drug users in confined settings in China should be considered.

Surveys among drug users in closed settings in China and elsewhere rarely inquire about drug use and injecting behaviours whilst incarcerated. This is a sensitive issue for both prison authorities and prisoners, who may fear retribution by disclosing drug use practices while incarcerated. Nevertheless, we found that if the survey is conducted in a private setting without the presence of authorities, the respondent is aware that data will remain confidential from authorities, and that their responses will be aggregated to maintain anonymity, then this reduces prisoners’ fears of retribution. In China, health researchers have long neglected researching prisoner health, giving little consideration to the issue when designing studies or programmes for drug users [17]. Some argue that prison surveys dealing with sensitive topics like drug use in closed settings may not produce accurate results from detainees. However, we found that 98% men and 96% women detainees who completed this survey reported that they were ‘honest’ in all or most of their answers.

During the study, Chinese counterparts informed researchers that RTLCs, as mandated by the Narcotics Control Law of the People’s Republic of China, effective 1 June 2008 [29], would be engaging in the process of institutional reform transforming themselves into ‘drug rehabilitation centres’. Laws had been passed to reform drug treatment and rehabilitation, in effect, eliminating compulsory detoxification centres and re-education-through-labour camps and integrating both into the “isolated compulsory drug rehabilitation” or compulsory isolation centres [29]. This law takes a more humanitarian approach to drug addiction; drug users previously identified as lawbreakers are now regarded as patients and victims who need to be medically treated, educated and integrated back into society [29].

“… The state takes various kinds of measures to educate, save and help addicts to shake off the obsession of drugs. Drug addicts shall get drug rehabilitation treatment”.

(Chapter 4, Article 31) [29]

The law also provides details of how compulsory isolation centres should treat their patients/detainees:

“…An isolated compulsory drug rehab center shall provide necessary nursing and treatment for seriously disabled or sick drug addicts; take necessary measures for isolation or treatment for drug addicts with contagious disease; and take corresponding protective and restraint measures for drug addicts who may injure themselves. Managerial personnel of isolated compulsory drug rehab centers may not physically punish, abuse or insult drug addicts…”.

(Chapter 4, Article 44) [29]

Institutional reform is slow and some RTLCs recently have partially dismantled their institutions [30]. For the RTLCs in our survey, almost three years had passed since the new laws were legislated before the survey was conducted, and each RTLC visited had not dismantled and reformed themselves into “compulsory isolation centres”; nor did they provide methadone or other opiate substitution treatment to drug user detainees. Drug treatment in China is primarily methadone delivered by government health agencies to drug users in the community [31,32].

### Limitations

Limitations of the present study include the reliance on self-reported drug use and the possibility that participants provided socially desirable responses. However, the findings in regard to lifetime drug use and sex work suggest that this is unlikely, although few reported drug use inside RTLCs and CDCs. A further limitation was that the selection of RTLC sites and the study sampling strategy were largely controlled by the Re-education-Through-Labour Administration Bureau in the region but with reference to suggestions from study epidemiologists in China. However, from our preliminary investigations, we did not detect any major differences in the characteristics of detainees of the RTLCs in the region between those that participated in the study and those that did not. We included the women-only labour camp in the province in our study.

## 5. Conclusions

This study shows that a significant proportion of those in RTLC have a history of drug use and drugs-related incarceration, suggesting that RTLCs should offer expanded drug treatment and education services, even prior to planned institutional reforms. High rates of relapse to drug use after release highlight the need to address the medical aspects of drug dependence in closed settings. This will require a shift from a primarily punitive approach to drug dependence to one that incorporates medical treatment. HIV prevalence among detainees is comparable to that of community IDUs. Repeat incarcerations in CDTCs/RTLCs were associated with higher risks of HIV infection.

## References

[1] Mathers B.M., Degenhardt L., Ali H., Wiessing L., Hickman M., Mattick R.P., Myers B., Ambekar A., Strathdee S.A. (2010). HIV prevention, treatment, and care services for people who inject drugs: A systematic review of global, regional, and national coverage. Lancet.

[2] (2012). China’s Annual Report on Drugs.

[3] Freudenberg N. (2001). Jails, prisons, and the health of urban populations: A review of the impact of the correctional system on community health. J. Urban Health.

[4] Vescio M.F., Longo B., Babudieri S., Starnini G., Carbonara S., Rezza G., Monarca R. (2008). Correlates of hepatitis C virus seropositivity in prison inmates: A meta-analysis. J. Epidemiol. Community Health.

[5] Calzavara L.M., Burchell A.N., Schlossberg J., Myers T., Escobar M., Wallace E., Major C., Strike C., Millson M. (2003). Prior opiate injection and incarceration history predict injection drug use among inmates. Addiction.

[6] Kang S.Y., Deren S., Andia J., Colon H.M., Robles R., Oliver-Velez D. (2005). HIV transmission behaviors in jail/prison among puerto rican drug injectors in New York and Puerto Rico. AIDS Behav..

[7] Martin R.E., Gold F., Murphy W., Remple V., Berkowitz J., Money D. (2005). Drug use and risk of bloodborne infections: A survey of female prisoners in British Columbia. Can. J. Public Health.

[8] Zamani S., Farnia M., Torknejad A., Alaei B.A., Gholizadeh M., Kasraee F., Ono-Kihara M., Oba K., Kihara M. (2010). Patterns of drug use and HIV-related risk behaviors among incarcerated people in a prison in Iran. J. Urban Health.

[9] Indig D., Topp L., Ross B., Mamoon H., Border B., Kumar S., McNamara M. (2010). 2009 NSW Inmate Health Survey: Key Findings Report.

[10] Thaisri H., Lerwitworapong J., Vongsheree S., Sawanpanyalert P., Chadbanchachai C., Rojanawiwat A., Kongpromsook W., Paungtubtim W., Sri-Ngam P., Jaisue R. (2003). HIV infection and risk factors among Bangkok prisoners, Thailand: A prospective cohort study. BMC Infect. Dis..

[11] Dolan K., Kite B., Black E., Aceijas C., Stimson G.V., Reference Group on HIV/AIDS Prevention and Care among Injecting Drug Users in Developing and Transitional Countries (2007). HIV in prison in low-income and middle-income countries. Lancet Infect. Dis..

[12] (2007). Interventions to Address HIV in Prisons: Needle and Syringe Programmes and Decontamination Strategies.

[13] (2003). Re-Education through Labor Drug Detoxification Regulations. http://www.gov.cn/gongbao/content/2003/content_62443.htm.

[14] Zhang L., Liu J., Huang K. (2011). The role of criminal justice system in treating drug abusers: The Chinese experience. J. Subst. Abuse Treat..

[15] Biddulph S., Xie C. (2011). Regulating drug dependency in China: The 2008 PRC drug prohibition law. Brit. J. Criminol..

[16] (2008). Narcotics Control Law of the People’s Republic of China. http://www.gov.cn/ziliao/flfg/2007-12/29/content_847311.htm.

[17] Cohen J.E., Amon J.J. (2008). Health and human rights concerns of drug users in detention in Guangxi Province, China. PLoS Med..

[18] Wang L., Wang N., Wang L., Li D., Jia M., Gao X., Qu S., Qin Q., Wang Y., Smith K. (2009). The 2007 estimates for people at risk for and living with HIV in China: Progress and challenges. J. Acquir. Immune Defic. Syndr..

[19] Chinese Ministry of Health (2012). China AIDS Response Progress Report 2012.

[20] Bao Y.P., Liu Z.M., Lu L. (2010). Review of HIV and HCV infection among drug users in China. Curr. Opin. Psychiatr..

[21] Hu Y., Liang S., Zhu J., Qin G., Liu Q., Song B., Wang Q., Wei D., Zhang L., Qian H.-Z. (2011). Factors associated with recent risky drug use and sexual behaviors among drug users in southwestern China. AIDS Clin. Res..

[22] Wu J., Huang J., Xu D., Lu C., Deng X., Zhou X. (2010). Infection status and risk factors of HIV, HBV, HCV, and syphilis among drug users in Guangdong, China—A cross sectional study. BMC Public Health.

[23] Lau J.T., Feng T., Lin X., Wang Q., Tsui H.Y. (2005). Needle sharing and sex-related risk behaviours among drug users in Shenzhen, a city in Guangdong, southern China. AIDS Care.

[24] Huang K., Zhang L., Liu J. (2011). Drug problems in contemporary China: A profile of Chinese drug users in a metropolitan area. Int. J. Drug Policy.

[25] Huang J., Jiang J., Li J.Z., Yang X., Deng W., Abdullah A.S., Qin B., Upur H., Zhong C., Wang Q. (2013). Prevalence and correlates of sexual risk behaviors among drug users in western China: Implications for HIV transmission. AIDS Res. Hum. Retroviruses.

[26] Butler T., Levy M., Dolan K., Kaldor J. (2003). Drug use and its correlates in an Australian prisoner population. Addict. Res. Theory.

[27] Yang L., Li J., Zhang Y., Li H., Zhang W., Dai F., Ren Z., Qi G., Cheng W. (2008). Societal perception and support for methadone maintenance treatment in a Chinese province with high HIV prevalence. Am. J. Drug Alcohol Abuse.

[28] Zhang L., Chow E.P., Zhuang X., Liang Y., Wang Y., Tang C., Ling L., Tucker J.D., Wilson D.P. (2013). Methadone maintenance treatment participant retention and behavioural effectiveness in China: A systematic review and meta-analysis. PLoS One.

[29] Narcotics Control Law of the People’s Republic of China Effective date 1 June 2008. Standing Committee of the National People’s Congress. http://www.lawinfochina.com/display.aspx?lib=law&id=6604&CGid=.

[30] Guangzhou to Release Labor Camp Detainees. China Digital Times. http://chinadigitaltimes.net/2013/09/guangzhou-release-remaining-labor-camp-detainees/.

[31] Wang W., Xu J., Jiang S., Yang K., Meng Z., Ma Y., Li M., Zhang X., Shao Y., Zhang F. (2011). The dynamic face of HIV-1 subtypes among men who have sex with men in Beijing, China. Curr. HIV Res..

[32] Lu L., Zhao D., Bao Y.P., Shi J. (2008). Methadone maintenance treatment of heroin abuse in China. Am. J. Drug Alcohol Abuse.

